# A closer look at visually guided saccades in autism and Asperger’s disorder

**DOI:** 10.3389/fnint.2012.00099

**Published:** 2012-11-07

**Authors:** Beth P. Johnson, Nicole J. Rinehart, Nicole Papadopoulos, Bruce Tonge, Lynette Millist, Owen White, Joanne Fielding

**Affiliations:** ^1^Centre for Developmental Psychiatry and Psychology, School of Psychology and Psychiatry, Monash UniversityMelbourne, VIC, Australia; ^2^Department of Neurology, Royal Melbourne HospitalParkville, VIC, Australia

**Keywords:** autism, Asperger’s disorder, saccades, eye movements, Verbal Comprehension Index

## Abstract

Motor impairments have been found to be a significant clinical feature associated with autism and Asperger’s disorder (AD) in addition to core symptoms of communication and social cognition deficits. Motor deficits in high-functioning autism (HFA) and AD may differentiate these disorders, particularly with respect to the role of the cerebellum in motor functioning. Current neuroimaging and behavioral evidence suggests greater disruption of the cerebellum in HFA than AD. Investigations of ocular motor functioning have previously been used in clinical populations to assess the integrity of the cerebellar networks, through examination of saccade accuracy and the integrity of saccade dynamics. Previous investigations of visually guided saccades in HFA and AD have only assessed basic saccade metrics, such as latency, amplitude, and gain, as well as peak velocity. We used a simple visually guided saccade paradigm to further characterize the profile of visually guided saccade metrics and dynamics in HFA and AD. It was found that children with HFA, but not AD, were more inaccurate across both small (5°) and large (10°) target amplitudes, and final eye position was hypometric at 10°. These findings suggest greater functional disturbance of the cerebellum in HFA than AD, and suggest fundamental difficulties with visual error monitoring in HFA.

## INTRODUCTION

Autism and Asperger’s disorder (AD) are pervasive developmental disorders that share disturbances in social interaction and communication, as well as repetitive and stereotyped behaviors and interests (American Psychiatric Association, 2000). AD is currently differentiated from autism by the absence of clinically significant delays in language (single words used by age 2 years, communicative phrases used by age 3 years), and no delays in cognitive development (American Psychiatric Association, 2000). In addition to the core symptoms associated with autism and AD, motor impairments have been consistently reported in these groups (Fournier et al., 2010) impacting postural control (Gepner and Mestre, 2002), fine motor (Cartmill et al., 2009), upper limb (Martineau et al., 2004; Papadopoulos et al., 2012; Rinehart et al., 2006b,c), and ocular motor control (Takarae et al., 2004; Nowinski et al., 2005; Stanley-Cary et al., 2011). Green et al. (2002) also found that motor performance on the Movement Assessment Battery for Children, which assesses manual dexterity, aiming and catching, and balance, was significantly correlated with IQ across children with autism and AD (Green et al., 2009). However, few studies have directly compared motor functioning in autism and AD, although limited findings have revealed differences between the motor profiles two groups in studies of gait (Rinehart et al., 2006b; Nayate et al., 2011) and upper limb function (Rinehart et al., 2001, 2006a; Papadopoulos et al., 2012). The next revision of the *Diagnostic and Statistical Manual of Mental Disorders *will see the amalgamation of**autism and AD into a single *autism spectrum disorders *category, however, determining whether a history of language and cognitive delay is associated with additional motor symptoms is essential to establishing a comprehensive understanding of the symptomatology of these disorders, and for the development of appropriately tailored interventions for autism and AD.

Current neuroanatomical evidence has also indicated that involvement of the cerebellum across autism and AD. In autism, abnormalities are consistently reported within the cerebellar vermis lobules VI–VII, also known as the ocular motor vermis (Courchesne et al., 1994a; Townsend et al., 1996; Allen and Courchesne, 2003). Efferents of vermis lobules VI–VII project predominantly to the fastigial nuclei, one of the three output nuclei of the cerebellum (Scudder, 2002). Smaller neurons and reduced cell numbers have also been reported in the fastigial nuclei in high-functioning autism (HFA; Bauman, 1991). However, the role of the cerebellum in the context of AD is poorly understood. Although the primary site of pathology within the cerebellum is unclear in AD, there is a general consensus the degree of cerebellar disruption is more limited in AD than in autism (Lotspeich et al., 2004; Bauman and Kemper, 2005; Catani et al., 2008; Yu et al., 2011). The cerebellum, in particular vermis lobules VI–VII and the fastigial nuclei, are crucial to the control of eye movements (Ohtsuka and Nodu, 1995; Barash et al., 1999), and several investigations of functional impairment associated with cerebellar abnormalities have used ocular motor paradigms in autism and AD (Takarae et al., 2004; Nowinski et al., 2005; Stanley-Cary et al., 2011).

Well timed eye movements are essential for accurate visual perception (Hernandez et al., 2008) and attention (Courchesne et al., 1994b) as well as enhancing the precision motor actions where the eye and hand are coupled, such as reaching and grasping or catching a ball (Cotti et al., 2007). Visually guided (or reflexive) saccades, which are initiated in response to novel exogenous stimuli, are of particular interest for comparing autism and AD as they eliminate many confounds relating to differences in cognitive and language development history between autism and AD. Previous studies of visually guided saccades in autism reported hypometric saccades and more variable error (scatter) of saccade endpoints (Rosenhall et al., 1988; Takarae et al., 2004; Luna et al., 2007; Stanley-Cary et al., 2011). In comparisons of visually guided saccades between autism and AD, AD shows a tendency toward hypometric primary saccades but no evidence of increased saccade variability (Takarae et al., 2004). Fundamental abnormalities in reflexive saccades may conceivably have detrimental, downstream consequences for several features of autism and AD, such as cognition, language acquisition, attention, or visuomotor coordination (Brenner et al., 2007).

To date, examination of visually guided saccades in HFA and AD have used relatively elementary assessments of the saccadic profile, such as latency, amplitude, peak velocity, and duration (Minshew et al., 1999; Goldberg et al., 2000). Extensive investigations of the role of the cerebellum in eye movements in both humans and non-human primates have demonstrated that assessing saccade dynamics, such as velocity skewness as well as the relationship between saccade metrics and dynamics, such as examining the main sequence (relationship between peak velocity and amplitude) and Q-ratio (relationship between peak velocity and mean velocity), is a sensitive way by which to fully characterize the integrity of the cerebellar vermis and fastigial nuclei network (Robinson et al., 1993; Ohtsuka and Nodu, 1995; Takagi et al., 1998; Collins et al., 2008; Federighi et al., 2011). Examination of final eye position (FEP), as well as the primary saccade amplitude, can also provide insight regarding the accuracy of corrective saccades. Moreover, full characterization of saccadic profile has been shown to be sensitive in discerning autism and AD in volitional saccade paradigms (Stanley-Cary et al., 2011). As reflexive, visually guided saccades often form the basis of comparison for higher order, volitional saccade tasks, thorough characterization of the metrics, and dynamics of reflexive saccades in autism and AD is essential.

The aim of this study was to further characterize visually guided saccade metrics and dynamics in individuals with HFA and AD and determine whether ocular motor deficits are associated with standardized measures of cognitive and motor performance. Firstly, we aimed to establish a complete description of saccade metrics and dynamics in children with HFA and AD, and determine whether these remained constant over saccade amplitude. Additionally, we sought to clarify whether variability of saccade accuracy was due to poor spatial encoding, as evidenced by a disrupted relationship between saccade latency and accuracy, or inherent variability of eye movements. It was hypothesized that children with HFA would show greater cerebellar-type ocular motor deficits relative to children with AD.

## MATERIALS AND METHODS

### PARTICIPANTS

This study was approved by Monash University and Southern Health Human Research Ethics Committees. Parents of participants provided informed consent prior to the commencement of the study, and written assent was provided by the participants in accordance with the Declaration of Helsinki.

Thirty-seven children aged between 9 and 14 years participated in the study: 10 with HFA (all male), 15 with AD (10 males: 5 females) and 12 typically developing (8 males: 4 females) children (see **Table 1** for a summary of participant characteristics). Children with HFA and AD were recruited from private pediatricians in Melbourne, Victoria and the Autism Victoria database. Reports from pediatricians were reviewed to ensure that all children were diagnosed according to Diagnostic and Statistical Manual of Mental Disorders – 4th edition, revised (DSM-IV-TR; American Psychiatric Association, 2000) criteria for autistic disorder. Further diagnostic information was gathered using the Social Responsiveness Scale (SRS), Developmental Behavior Checklist – Parent Version (DBC-P), structured parent interviews, direct child observations and information from teachers and other therapists involved in the assessment process. The DBC-P has good psychometric properties, includes five subscales (disruptive/antisocial, self-absorbed, communication disturbance, anxiety, and social relating), and provides an autism screening algorithm (autism-related items are weighted and collated to calculate an overall risk index; Brereton et al., 2002; Witwer and Lecavalier, 2007). Participants were excluded if they were suffering from any co-morbid neurological (e.g., tuberous sclerosis), genetic (e.g., Fragile X syndrome), or psychiatric diagnosis (e.g., Tourette’s syndrome). No children in the HFA or AD groups were reported to have been taking any type of medication.

**Table 1:** Participant characteristics.

	HFA		AD		TD	
	Mean	SD	Mean	SD	Mean	SD
Age (months)	134.90	17.28	153.93	42.33	139.58	18.92
Full scale IQ	95.90	15.22	104.20	14.01	108.50	11.09
Verbal Comprehension Index	99.00	18.02	107.87	15.62	108.92	15.02
Perceptual Reasoning Index	102.10	21.00	104.00	13.56	104.33	13.20
**MABC-2**						
Total score	6.90*	**3.38**	7.73	3.43	10.78	2.64
Manual dexterity	7.10	3.18	7.00	2.71	8.92	1.83
Aiming and catching	8.40*	**2.55**	**8.46^‡^**	**3.50**	12.75	3.39
Balance	7.80*	**3.82**	10.00	3.83	10.92	2.71

*HFA, high-functioning autism; AD, Asperger’s disorder; TD, typically developing; SD, standard deviation; MABC-2, Movement Assessment Battery for Children – 2nd edition.*

* HFA vs TD *p* < 0.05; ^‡^AD vs TD *p* < 0.05.

Typically developing boys were recruited from community-wide organizations. The presence of motor impairment was screened for using the Movement Assessment Battery for Children – 2nd edition (MABC-2), and normal behavioral functioning was screened for using the DBC-P and SRS in order to exclude the presence of autism, AD, or other previously listed psychiatric diagnosis.

Motor skills of all children were assessed using the MABC-2, which has previously been used to assess motor performance in children with HFA and AD (Green et al., 2002). The MABC-2 consists of eight items grouped in three sections: manual dexterity, ball skills and balance, with age dependent items used for each section. No TD participants fell in the “definite motor impairment range” as defined by the MABC-2 guidelines.

All children completed the Wechsler Intelligence Scale for Children – 4th edition (WISC-IV). Analysis of variance (ANOVA) was used to compare age and IQ scores between the three groups (see **Table 1** for participant characteristics). The groups did not differ on age [F(2,36) = 1.36, *p* = 0.27], FSIQ [F(2,36) = 2.22, *p* = 0.12], VCI [F(2,36) = 1.12, *p* = 0.30], or PRI [F(2,36) = 0.063, *p* = 0.94].

### APPARATUS

Eye movements were recorded at 500 Hz using a head-mounted Eyelink II video-oculographic eye tracking system, which has a sensitivity of <0.01°. Stimuli were generated using Experiment Builder v1.10 (SR Research Ltd., Mississauga, Canada) and displayed on a 22′ CRT monitor with a screen refresh rate of 100 Hz. Stimuli were presented on a black background and comprised a green target in the shape of a cross (30 mm × 30 mm) which was presented centrally, 5° or 10° from center in either hemifield, and a white centrally positioned square ring (10 mm × 10 mm) which served as the refixation stimulus.

Prior to testing, participants were shown the equipment and given time to familiarize themselves with the head-mounted cameras and ocular motor testing procedure. Participants were seated 840 mm directly in front of the monitor with their heads stabilized using a custom-made chin and head rest. Whole body movements, which can also introduce instability in eye movement recording, were controlled with use of feet and arm rests, and high backed chair to support the shoulders and upper body.

Eye movement data were analyzed off line using a customized MATLAB program developed in our laboratory.

### PROCEDURE

The task included 32 trials (16 left, 16 right, balanced for 5° and 10° steps). Participants fixated on a centrally positioned target. After a random time period of either 1250 or 1600 ms, the central target was extinguished and concomitantly a peripheral target appeared. The peripheral target was extinguished after 1500 ms and a refixation stimulus appeared for 150 ms to redirect gaze back to the center in preparation for the next trial.

### DATA ANALYSIS

Trials were excluded from further analysis if they exhibited (1) blinks prior to 100 ms of the target onset or during the primary saccade, (2) unstable fixation on the centrally presented target, or (3) small saccades with amplitude <3°.

First saccade gain, defined as [first saccade amplitude/target amplitude], was used as a measure of saccade dysmetria. The FEP was defined as the fixation position reached following the primary saccade plus any corrective saccades; FEP gain was defined as [FEP/target amplitude].

Variable error was calculated as the standard deviation of gain. This was used as a measure of the consistency of saccade endpoints, with higher values indicating reduced movement consistency.

Mean absolute percentage error (MAPE), calculated as [(eye position - target position)/target position] × 100, was used as a measure of absolute movement error, irrespective of direction.

Saccade latency was defined as [target onset - saccade onset]. [Latency/first saccade gain] was used as measures of reaction time and to assess whether saccade accuracy changed with processing time (Cohen et al., 2007).

Saccade dynamics were used to determine whether there was any change in the waveform relationships across amplitude, which can be indicative of disruption to cerebellar-brainstem motor circuitry deficits. The time from saccade onset to peak velocity, and from peak velocity to 0, were used to calculate the velocity skewness [time to peak velocity/time from peak velocity to 0]. Q-ratio [Peak velocity/Mean velocity] and main sequence [peak velocity/first saccade amplitude] were also assessed.

### STATISTICAL ANALYSES

Data were analyzed with SPSS v.18.0. Mixed model ANOVAs with target direction as the within subjects variable and group as the between subjects variable revealed no significant interaction between group and target direction for any dependent variable. All data were therefore collapsed across direction for group analyses using a series of one-way ANOVAs.

Peak velocity/mean velocity ratio, variable error of first saccade gain and FEP MAPE each violated Levene’s test of equality of variance (*p* < 0.05), therefore in these instances Brown–Forsythe test was used for comparison between groups. *Post hoc* Tukey’s HSD tests, or Games–Howell tests in instances where homogeneity of variance was violated, were used to investigate group differences.

## RESULTS

### PRIMARY SACCADE METRICS

Primary saccade gain and variable error of primary saccade gain did not differ between groups for 5° or 10° target amplitudes.

Children with HFA, but not AD, showed increased MAPE at both 5° and 10° target amplitudes relative to controls. There was a significant difference in MAPE at 5° [F(2,36) = 5.04, *p* = 0.012], with *post hoc* analysis revealing significant differences between HFA and TD groups (*p* = 0.011) and HFA and AD groups (*p* = 0.050), but not between AD and TD groups. There was also a significant difference in primary saccade MAPE at 10° [F(2,36) = 4.19, *p* = .024], with *post hoc* analysis revealing significant differences between HFA and TD groups (*p* = 0.020) and trend toward significance between HFA and AD groups (*p* = 0.054), but not between AD and TD groups.

### FINAL EYE POSITION METRICS

Children with HFA showed hypometric FEP at 10° target amplitudes, but not 5° target amplitudes (**Table 2**). There was a significant difference in FEP between groups at 10° [F(2,36) = 6.00, *p* = 0.006], with *post hoc* analysis revealing significant differences between HFA and TD groups (*p* = 0.006) and HFA and AD groups (*p* = 0.026), but not between AD and TD groups (*p* = 0.73). FEP gain did not differ between groups for the 5° targets [F(2,36) = 0.52, *p* = 0.60].

**Table 2:** Group means and standard deviations for saccade latency and metrics.

	HFA		AD		TD	
Mean	SD	Mean	SD	Mean	SD	Mean
**Saccade metrics**						
***First saccade gain***						
5°	0.97	0.10	0.96	0.10	0.95	0.08
10°	0.87	0.08	0.90	0.06	0.91	0.04
***Variable error of first saccade gain***						
5°	0.17	0.11	0.15	0.08	0.12	0.04
10°	0.14	0.09	0.13	0.04	0.10	0.01
***First saccade MAPE***						
5°	16.64*	7.68	14.21	5.06	10.15	1.96
10°	18.19*^‡^	**7.26**	12.94	5.00	11.17	3.60
**Final eye position gain**
5°	1.04	0.13	1.02	0.06	1.00	0.05
10°	0.95*	0.06	1.00	0.03	1.01	0.04
***Variable error of final eye position gain***						
5°	0.19	0.15	0.14	0.09	0.10	0.06
10°	0.17*	0.13	0.09	0.05	0.09	0.04
***Final eye position MAPE***						
5°	13.07	8.27	9.37	4.25	8.33	3.41
10°	11.56	7.86	6.13	2.29	6.70	2.03
***Latency***						
5°	173.16	21.65	181.35	30.97	170.38	17.65
10°	175.16	18.59	173.56	25.34	176.44	21.97
***Latency/saccade gain***						
5°	208.37	30.7	198.36	28.8	200.39	30.76
10°	183.76	28.58	191.21	34.03	184.82	28.22

*HFA, high-functioning autism; AD, Asperger’s disorder; TD, typically developing; SD, standard deviation; MAPE, mean absolute percentage error.*

* HFA vs TD *p* < 0.05

‡ HFA vs AD *p* = 0.054.

Children with HFA also showed greater variability in FEP at large target amplitudes, but not smaller target amplitudes (**Table 2**). There was a significant difference in FEP variable error between groups at 10° [F(2,36) = 4.25, *p* = 0.02], with *post hoc* analysis revealing significant differences between HFA and TD groups (*p* = 0.036) and HFA and AD groups (*p* = 0.038), but not between AD and TD groups (*p* = 0.98). Variable error of FEP gain did not differ between groups for the 5° targets.

There was no significant difference in FEP MAPE between groups at 10° or 5° target amplitudes.

### LATENCY

There was no difference between groups with respect to latency or latency/saccade gain ratio for saccades made to either 5° or 10° target amplitudes.

### SACCADE DYNAMICS

There was no difference in the velocity profile of saccades as evidenced by no between-group differences in saccade duration, peak velocity, time to peak velocity, time from peak velocity to 0, velocity skewness, peak velocity/mean velocity ratio, or main sequence at either 5° or 10° target amplitudes (**Table 3**).

**Table 3:** Group means and standard deviations for saccade dynamics.

HFA			AD		TD	
	Mean	SD	Mean	SD	Mean	SD
**Saccade duration**						
5°	31.48	5.21	32.78	3.44	30.83	3.56
10°	41.64	5.28	43.47	5.07	42.74	3.66
**Peak velocity**						
5°	270.14	45.32	266.05	31.89	278.85	54.83
10°	362.27	49.33	372.35	41.13	377.18	49.33
**Time to peak velocity (ms)**						
5°	14.02	2.63	14.47	2.70	12.61	1.55
10°	17.41	3.94	18.13	2.94	17.00	2.41
**Time from peak velocity to 0 (ms)**						
5°	17.45	3.74	18.30	2.27	18.21	3.60
10°	24.22	3.28	25.33	4.69	25.73	3.99
**Velocity skewness**						
5°	0.86	0.18	0.84	0.19	0.77	0.20
10°	0.75	0.19	0.76	0.19	0.71	0.17
**Peak velocity/mean velocity**						
5°	1.61	0.07	1.65	0.08	1.66	0.14
10°	1.63	0.18	1.63	0.08	1.63	0.12
**Main sequence**						
5°	56.44	8.06	55.95	8.08	59.27	9.03
10°	41.93	5.94	42.1	5.17	41.84	5.33

*Main sequence, peak velocity/amplitude; HFA, high-functioning autism; AD, Asperger’s disorder; TD, typically developing; SD, standard deviation.*

## DISCUSSION

In the present study, we sought to characterize the profile of reflexive saccade metrics and dynamics in children with HFA and AD. Our results confirmed previous reports of hypometria at large saccade amplitudes in children with HFA (Takarae et al., 2004; Stanley-Cary et al., 2011), and extended these findings to reveal inaccurate saccades at smaller amplitudes. Although primary saccades were more variable and hypometric in HFA, there was no evidence of associated changes to saccade dynamics. These subtle motor impairments are comparable to those seen in other motor modalities in HFA, such as gait (Rinehart et al., 2006c; Nayate et al., 2011) and upper limb function (Martineau et al., 2004; Papadopoulos et al., 2012).

The networks that underpin initiation and optimization of visually guided saccades can be conceptualized as two complimentary functional loops (Pierrot-Deseilligny et al., 1991, 1995; Scudder, 2002; Quaia et al., 2005). The first loop involves the visual cortex, parietal eye fields (PEFs), superior colliculus (SC), and brainstem pre-motor areas (Pierrot-Deseilligny et al., 1995; Gaymard et al., 2003). The PEFs, which receive input from the visual cortex, integrate visuospatial information and generate a motor command in response to the sudden appearance of a target within the visual field (Gaymard et al., 2003). The motor command from the PEF is sent to pontine pre-motor nuclei in the brainstem via the SC. Abnormalities of the parieto-collicular pathway in visually guided saccades classically manifest as disturbances in saccade latency: poor visuospatial integration is associated with decreased saccade accuracy (Scialfa and Joffe, 1998; Cohen et al., 2007), while lesions of the PEF result in increased reflexive saccade latencies (Lynch and McLaren, 1989; Gaymard et al., 2003). We did not find any evidence of disrupted latency, or altered latency/saccade gain relationship suggestive of impairment of visuospatial attention (Cohen et al., 2007), which is consistent with previous findings for visually guided saccades in HFA and AD (Minshew et al., 1999; Takarae et al., 2004).

The second loop, which refines saccade amplitude and minimizes variability in response to visual error, involves the SC, cerebellar oculomotor vermis (lobules VI–VII), fastigial nucleus, and brainstem pre-motor nuclei (Robinson et al., 1993; Scudder, 2002; Quaia et al., 2005). In this loop, a copy of the motor command arising from the SC, which specifies the velocity and amplitude of a saccade, is sent via the nucleus reticularis tegmenti pontis to the cerebellar vermis lobules VI–VII (Scudder, 2002; Scudder et al., 2002). The error signal, which is the difference between the fovea and visual target after an initial dysmetric saccade is also to vermis lobules VI–VII via the inferior olive (Soetedjo et al., 2008). The cerebellar oculomotor vermis projects to the caudal region of the fastigial nucleus, which in turn, projects back to the pontine pre-motor nuclei, as well as the thalamus, basal ganglia, and cortical regions (Scudder, 2002). The cerebellar vermis lobules VI–VII and caudal fastigial nucleus are thought to be critical in fine-tuning saccade amplitude and dynamics, and minimize saccade error via the direct modulation of pre-motor circuitry (Noto and Robinson, 2001; Scudder, 2002; Xu-Wilson et al., 2009). It is this second loop that has been proposed to result in increased variability of saccade gain in HFA (Takarae et al., 2004; Stanley-Cary et al., 2011); findings from our study support this.

In typically developing children and adults, saccade endpoint accuracy changes over amplitude, with larger saccade eccentricities associated with greater hypometria (Fioravanti et al., 1995; Ploner et al., 2004; Irving et al., 2006). In the present study TD children conformed with these findings, performing hypometric to larger target amplitudes (10°), but not smaller amplitudes (5°). By contrast, children with HFA not only were more hypometric than TD children at the 10°, but first saccade gain was also more variable across both small and large target amplitudes. We found no evidence of additional disturbances of the velocity profile, or relationship between saccade metrics and dynamics in HFA or AD. That primary saccade accuracy was more variable in HFA, without an accompanying change in saccade dynamics is comparable to observations following cooling of the fastigial nuclei in non-human primates (Vilis and Hore, 1981). Cooling of the fastigial nucleus results in consistently hypometric saccades without a change to saccade dynamics. This was proposed to relate to impaired tuning of the internal representation of the eye muscles, such that ocular muscle strength is overestimated*.* This is thought to result in insufficient input to the brainstem pre-motor neurons from the fastigial nuclei, as the internal model predicts that the eye has achieved the correct target position sooner than it actually has (Vilis and Hore, 1981). Disruption to the oculomotor vermis–fastigial nuclei in autism network in HFA (Courchesne et al., 1988; Bauman, 1991; Allen and Courchesne, 2003), but not AD (Catani et al., 2008), may account for the functional differences in primary saccade accuracy in this group.

Of note is that FEP of visually guided saccades was hypometric and more variable at large saccade amplitudes in children with HFA, but not in AD. This is similar to findings previously found during volitional saccade paradigms comparing HFA and AD (Stanley-Cary et al., 2011). Despite ample time for visual feedback and correction, the displacement between the eye and target was not fully corrected for in children with HFA. This finding implies a fundamental deficit in online visual error monitoring and correction, consistent with more pronounced cerebellar disruption in HFA than AD. In addition to greater inaccuracy and greater variability of FEP, the HFA group also demonstrated poorer overall MABC-2 performance, as well as balance and aiming and catching, which further supports the proposal of greater functional disturbance of the cerebellum in HFA.

Deficits in performing accurate, ongoing corrective saccades may also have additional implications for accurate visual perception (Glazebrook et al., 2009) and coupling of eye–hand movements (Reina and Schwartz, 2003; Glazebrook et al., 2009) during motor tasks in children with HFA. Previous findings by Glazebrook et al. (2009), who examined the role of vision during manual aiming movements, found evidence of greater saccade amplitude variability as well as greater upper limb amplitude variability. Of key interest, however, is that children with AD performed more poorly on the aiming and catching component of the MABC-2, yet did not demonstrate greater saccade inaccuracy, or greater FEP inaccuracy. This finding implies that the difficulties children with AD have with aiming and catching may relate to upper limb or whole body coordination, or visuomotor integration, but not saccade accuracy. Moreover, it highlights that saccade accuracy and upper limb aiming accuracy, while coordinated, are relatively independent processes (Glazebrook et al., 2009), and further highlights that the underlying source of motor coordination difficulties may differ between HFA and AD. That saccade accuracy and motor abilities can be dissociated is of central importance when examining the elements that underpin visual and motor coordination impairments in these groups.

While groups in the present study did not differ on age, VCI, PRI, and full scale IQ measures, we did not control for the numbers of males and females between groups. There is no precedence for sex differences in saccade metrics or dynamics in typically developing children (Salman, 2006) or those with autism (Goldberg et al., 2000) however, this possibility cannot be eliminated. Our study was also limited by small samples size of groups, which may have hampered identification of abnormalities in the saccadic profile of AD, due to the subtlety of abnormality in the AD populations (Takarae et al., 2004, 2008).

## CONCLUSION

Ocular motor impairment associated neurodevelopmental abnormalities of the cerebellum are often more subtle than the symptoms classically associated with cerebellar damage (Salman et al., 2006; Tavano et al., 2007; Stanley-Cary et al., 2011), such as ataxia or lesioning (Barash et al., 1999; Fielding et al., 2010; Federighi et al., 2011). Our findings support a growing body of evidence implicating greater functional disturbance of the cerebellum in HFA than AD (Takarae et al., 2004; Nayate et al., 2005; Nowinski et al., 2005; Rinehart et al., 2006a,b; Stanley-Cary et al., 2011), consistent with current understanding of the neuropathology of these disorders (Abell et al., 1999; Bauman and Kemper, 2005; McAlonan et al., 2008, 2009; Yu et al., 2011). Our findings distinguish HFA from AD on the basis of ocular motor performance, which raises the concern that combining groups on the autism spectrum with different language and cognitive development histories may obscure important motor control features.

## Conflict of Interest Statement

The authors declare that the research was conducted in the absence of any commercial or financial relationships that could be construed as a potential conflict of interest.
